# Identification and characterization of the Non-race specific Disease Resistance 1 (NDR1) orthologous protein in coffee

**DOI:** 10.1186/1471-2229-11-144

**Published:** 2011-10-24

**Authors:** Jean-Luc Cacas, Anne-Sophie Petitot, Louis Bernier, Joan Estevan, Geneviève Conejero, Sébastien Mongrand, Diana Fernandez

**Affiliations:** 1 UMR 186 - IRD/CIRAD/UM2 Résistance des Plantes aux Bio-agresseurs, Institut de Recherche pour le Développement (IRD), BP64501, 34394 Montpellier Cedex 5, France; 2 Centre d'Étude de la Forêt, Université Laval, Québec (QC), G1V 0A6, Canada; 3 Plate-forme d'Histocytologie et d'Imagerie Cellulaire Végétale, Biochimie et Physiologie Moléculaire des Plantes-Développement et Amélioration des Plantes, INRA-CNRS-CIRAD, TA96/02 Avenue Agropolis, 34398 Montpellier, France; 4 Laboratoire de Biogenèse Membranaire (LBM), UMR 5200, CNRS-Université Victor Ségalen, Bordeaux 2, Case 92, 146 Rue Léo Saignat, 33076 Bordeaux Cedex, France

## Abstract

**Background:**

Leaf rust, which is caused by the fungus *Hemileia vastatrix *(Pucciniales), is a devastating disease that affects coffee plants (*Coffea arabica *L.). Disadvantages that are associated with currently developed phytoprotection approaches have recently led to the search for alternative strategies. These include genetic manipulations that constitutively activate disease resistance signaling pathways. However, molecular actors of such pathways still remain unknown in *C. arabica*. In this study, we have isolated and characterized the coffee *NDR1 *gene, whose *Arabidopsis *ortholog is a well-known master regulator of the hypersensitive response that is dependent on coiled-coil type R-proteins.

**Results:**

Two highly homologous cDNAs coding for putative NDR1 proteins were identified and cloned from leaves of coffee plants. One of the candidate coding sequences was then expressed in the *Arabidopsis *knock-out null mutant *ndr1-1*. Upon a challenge with a specific strain of the bacterium *Pseudomonas syringae *(DC3000::*AvrRpt2*), analysis of both macroscopic symptoms and *in planta *microbial growth showed that the coffee cDNA was able to restore the resistance phenotype in the mutant genetic background. Thus, the cDNA was dubbed *CaNDR1a *(standing for *Coffea arabica Non-race specific Disease Resistance 1a*). Finally, biochemical and microscopy data were obtained that strongly suggest the mechanistic conservation of the *NDR1*-driven function within coffee and *Arabidopsis *plants. Using a transient expression system, it was indeed shown that the CaNDR1a protein, like its *Arabidopsis *counterpart, is localized to the plasma membrane, where it is possibly tethered by means of a GPI anchor.

**Conclusions:**

Our data provide molecular and genetic evidence for the identification of a novel functional *NDR1 *homolog in plants. As a key regulator initiating hypersensitive signalling pathways, *CaNDR1 *gene(s) might be target(s) of choice for manipulating the coffee innate immune system and achieving broad spectrum resistance to pathogens. Given the potential conservation of *NDR1*-dependent defense mechanisms between *Arabidopsis *and coffee plants, our work also suggests new ways to isolate the as-yet-unidentified *R*-gene(s) responsible for resistance to *H. vastatrix*.

## Background

The genus *Coffea *includes about 120 species of subtropical/tropical woody perennial trees and shrubs (family *Rubiaceae*), of which at least two species are of worldwide agro-economic interest. Nearly 75% of world coffee production originates from *Coffea arabica *L., while about 20% comes from *C. canephora *Pierre ex A. Froehner (= *C. robusta*). Orange coffee leaf rust is considered to be one of the major plagues affecting *C. arabica *[1]. The fungus responsible for the disease, *Hemileia vastatrix *Berkeley & Broome, is widely spread throughout coffee-growing countries and can cause severe defoliation, resulting in substantial berry yield losses [1,2]. Furthermore, the two current approaches for restricting pathogen infection offer limited advantages. First, fungicide application, although cost-effective, does not always result in adequate disease control and, moreover, it has a negative environmental impact. Second, even though several varieties of coffee that are resistant to *H. vastatrix *have been used for introgression purposes [3,4], such alternatives are time-consuming and do not provide durable resistance due to the rapid co-evolution of races of the fungus that harbor new virulence genes [5]. Therefore, additional methods to control leaf rust in the fields are required.

*H. vastatrix *is an obligate biotrophic parasite belonging to the division Basidiomycetes, order Pucciniales [6]. Following urediospore germination on the abaxial leaf surface, hyphae grow and penetrate intercellular spaces of the mesophyll tissue through stomatal openings before differentiating intra-cellular feeding structures, or haustoria. In susceptible coffee plants, the successful pathogen can complete its dikaryotic cycle within three weeks following infection and reach the ultimate stage, which is characterized by the formation of a sporulating sorus. In resistant plants, hyphal invasion is rapidly sensed and arrested within 2-3 days [7,8]. Based on quantitative and Mendelian genetic studies [3,4], the occurrence of at least nine dominant resistance (*R*) genes in *Coffea *spp., and a similar number of fungal virulence genes, have been inferred. It is thus commonly accepted that the outcome of coffee/rust interactions, whether the plant resists pathogen attack (incompatibility) or develops disease (compatibility), relies on the gene-for-gene model [9], which has been recently amended [10]. Once delivered into coffee cells, *H. vastatrix *effector proteins, and the intracellular perturbations that they trigger, are supposed to be perceived by specific R-proteins. The recognition step promotes the launching of signaling defense pathway(s) and subsequent resistance. Alternatively, virulent rust races are believed to secrete effectors that escape or even counteract the host surveillance system, which allow for the highjacking of coffee cell metabolism and tissue colonization [11].

During incompatible interactions with biotrophic pathogens, the plant resistance phenotype results from the onset of a complex and multilayered-defense response, which is the so-called hypersensitive response or HR [12,13]. Although little is still known about the molecular mechanisms that govern resistance to *H. vastatrix*, several studies have advanced the case for the existence of a HR-like phenomenon in coffee plants. Resistant varieties that were inoculated with avirulent fungal strains displayed a morphotype that exhibits many HR characteristics. These include rapid host cell death, which is localized at the infection site and that is associated with fungal hyphae collapse [7,8], callose encasement of haustoria and subsequent cell wall lignification [8], early oxidative burst [14,15], and the activation of typical defense-related genes [16-18].

In previous work, we performed a suppression subtractive hybridization-based screening in *C. arabica *that had been challenged with *H. vastatrix *and identified a series of Expressed Sequence Tags (ESTs) that were regulated during compatible or incompatible interactions [16,19]. One of these ESTs shared a significant identity with the coding sequence of the *NON-RACE-SPECIFIC DISEASE RESISTANCE 1 *(*NDR1*) gene. Originally isolated in *Arabidopsis thaliana, NDR1 *encodes a small plasma membrane-resident protein, the deficiency of which was found to abolish HR and confer susceptibility to some fungal and bacterial pathogens carrying specific effector genes [20-22]. Notably, it has been established that *NDR1*-driven resistance is dependent on a specific subset of R-proteins (such as RPM1, RPS2 and RPS5) that are defined by the presence of a coiled-coil (CC) structure within their N-terminal parts [23]. From a mechanistic perspective, the best characterized example illustrating NDR1 function is the pathosystem involving strain DC3000::*AvrRpt2 *of the plant pathogenic bacterium *Pseudomonas syringae *pv. *tomato *(*Pst*). In this model, under resting conditions, AtNDR1 indirectly retains the RPS2 protein on the cytosolic side of the plasma membrane through its interaction with the RPM1-INTERACTING PROTEIN 4 (RIN4), thereby preventing HR activation [24]. Upon infection with *Pst*, the bacterial protease AvrRpt2 is secreted into the cytoplasm where it can cleave RIN4, releasing RPS2 and initiating a disease resistance signaling pathway [25].

In this study, we cloned two *C. arabica *candidate cDNAs for *NDR1 *and analyzed the deduced primary amino-acid sequences. Domain conservation and the high degree of homology between the coffee proteins and AtNDR1 led us to undertake a genetic complementation approach. Using the *Arabidopsis ndr1-1 *null mutant, we obtained genetic and molecular evidence that at least one of our candidate genes is a functional *NDR1 *ortholog. Both laser-confocal microscopy and biochemical analyses further suggested that the protein is likely to be attached to the plasma membrane via a glycosylphosphatidylinositol-anchor. Based on these data, the possibility that a NDR1-contingent mechanism could be invoked in *R*-gene-mediated resistance to *H. vastatrix *is discussed. The impact this result could have in the context of resistance improvement is also outlined.

## Results

### *Cloning and analysis of a novel NDR1 sequence homolog from *Coffea arabica

In previous work [19], we used a subtractive hybridization approach to identify genes involved in defense/resistance of coffee plants (*C. arabica L.*) to the orange rust fungus *H. vastatrix*. Of the 9 ESTs which were significantly up-regulated during HR, one displayed 43% identity with the canonical *NDR1 *coding sequence from *A. thaliana*. In this study, we focused our efforts on the coffee candidate for *NDR1 *gene and isolated two distinct full-length transcripts by nested RACE-PCR. The corresponding cDNAs were cloned as described in the '*Methods*' section (*CaNDR1a *[GenBank:DQ335596], *CaNDR1b *[GenBank:DQ335597]). Open reading frames differed from one another by only 3 single nucleotide positions with one of the substitutions being non-silent (F69L). Both sequences were predicted to encode proteins that were 214 amino acids long, which shared a calculated molecular weight of 23.8 kDa and an isoelectric point of 9.58.

**Figure 1:**
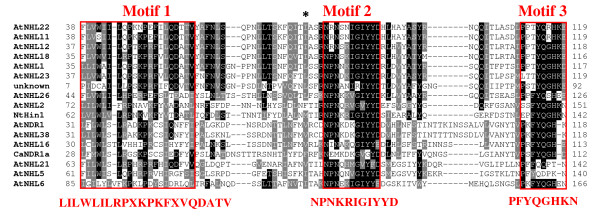
**The two coffee candidates for NDR1 protein belong to the NHL family**. Putative *Arabidopsis *orthologs of CaNDR1a/b proteins were identified by means of the BLAST algorithm using as queries the two deduced coffee amino-acid sequences. The retrieved sequences were aligned using version 2.0.10 of the Clustal X program [59] and the resulting alignment was then processed online at the BoxShade server (http://www.ch.embnet.org/software/BOX_form.html). The conserved region containing the three NHL motifs is presented. The position of the motifs is indicated with red lines and numbers. An asterik shows the position of the substituted amino-acid residue between the two coffee proteins (F69L). The full length sequence alignment can be found in Additional file 1. Accession numbers of the genes coding for the *Arabidopsis *proteins are as follows: NDR1 [AGI:At3g20600]; NHL1 [AGI:At3g11660]; NHL2 [AGI:At3g11650]; NHL5 [AGI:At1g61760]; NHL6, [AGI:At1g65690]; NHL11 [AGI:At2g35970]; NHL12 [AGI:At2g35960; NHL16 [AGI:At3g20610]; NHL18 [AGI:At3g52470]; NHL21 [AGI:At4g05220]; NHL22 [AGI:At4g09590]; NHL23 [AGI:At5g06330]; NHL26 [AGI:At5g53730]; NHL38 [AGI:At3g20590]; unknown, [AGI:At5g05657]. The accession number of the *Nicotiana tabacum *Hin1 coding sequence is GenBank: AB091429.1.

**Figure 2:**
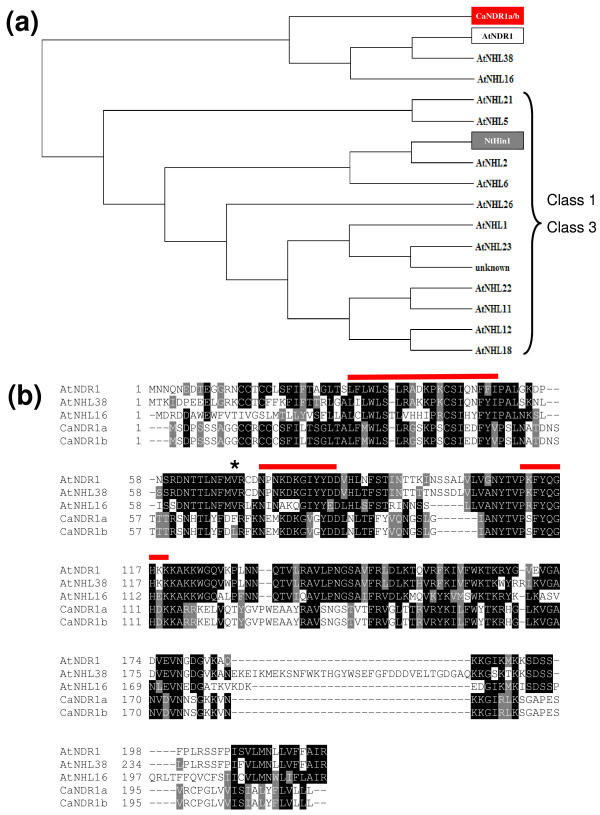
**NDR1, NHL16 and NHL38 are the closest *Arabidopsis *relatives of CaNDR1 proteins**. (a) Phylogenetic relationships between CaNDR1 proteins and their *Arabidopsis *relatives. The phylogenetic tree was built using the Phylowin freeware using the neighbor-joining method [60]. Sequence alignment was previously obtained using version 2.0.10 of the Clustal X program [59]. (b) Full length sequence alignment of CaNDR1a/b and the *Arabidopsis *protein NDR1, NHL16 and NHL38. Locations of the three NHL motifs within sequences are indicated with red lines above the alignment. The star indicates the amino acid residue substituted between both coffee NDR1 sequences. For sequence accession numbers, *see *legend of Figure 1.

Searching for *Arabidopsis *relatives of our proteins, we screened the GenBank database by means of the BLAST P algorithm [26] and retrieved 15 non-redundant hits. As expected, the best match appeared to be NDR1 with 42/61% identity/homology. Apart from an unknown sequence, all identified homologs had been previously described as members of the NDR1/HIN1-like (NHL) protein superfamily [27]. NHLs account for a vast class of plant defense-associated proteins that, within their N-terminal halves, contain two highly conserved peptide patterns (motifs 2 and 3) and a less conserved one (motif 1) [28]. Alignment of the proteins, along with the tobacco HIN1 for comparison, revealed the position of the three motifs within sequences (Figure 1; see also additional file 1 for full length sequence alignment). A phylogenetic analysis using solely the conserved region that is presented in Figure 1, and which encompasses the three NHL motifs, showed that CaNDR1a/b, NDR1, NHL38 and NHL16 formed a group that was distinct from other NHLs (Figure 2a,b). These data indicate that NDR1, NHL38 and NHL16 are the closest *Arabidopsis *relatives of CaNDR1a/b.

### *Ectopic expression of CaNDR1a in *Arabidopsis *ndr1-1 null mutant restores specific resistance to *Pseudomonas syringae *pv*. tomato *(DC3000::AvrRpt2)*

From our *in silico *analysis, the question arises as to whether the two identified coffee genes are functional homologs of *AtNDR1 *or code for distinct NHL counterparts. To answer this question, a genetic complementation approach was undertaken. Given the high degree of identity between the two predicted CaNDR1 amino-acid sequences, we decided to study CaNDR1a and expressed the corresponding ORF under the control of the *CaMV35S *promoter in the *Arabidopsis ndr1-1 *null mutant. Segregation analysis on a selective medium allowed for the isolation of single-locus, homozygous insertion lines (see additional file 2 for segregation results). T3 lines were then screened by RT-qPCR for high steady-state levels of transgene transcripts and three of them were selected for further experiments. The expression level of *CaNDR1a *in these lines, designated T3-1, T3-2 and T3-3, was respectively 92-, 190-, and 714-fold higher than that of the endogenous *AtNDR1 *gene, when compared to WT *Col-0 *plants grown under the exact same conditions.

**Figure 3:**
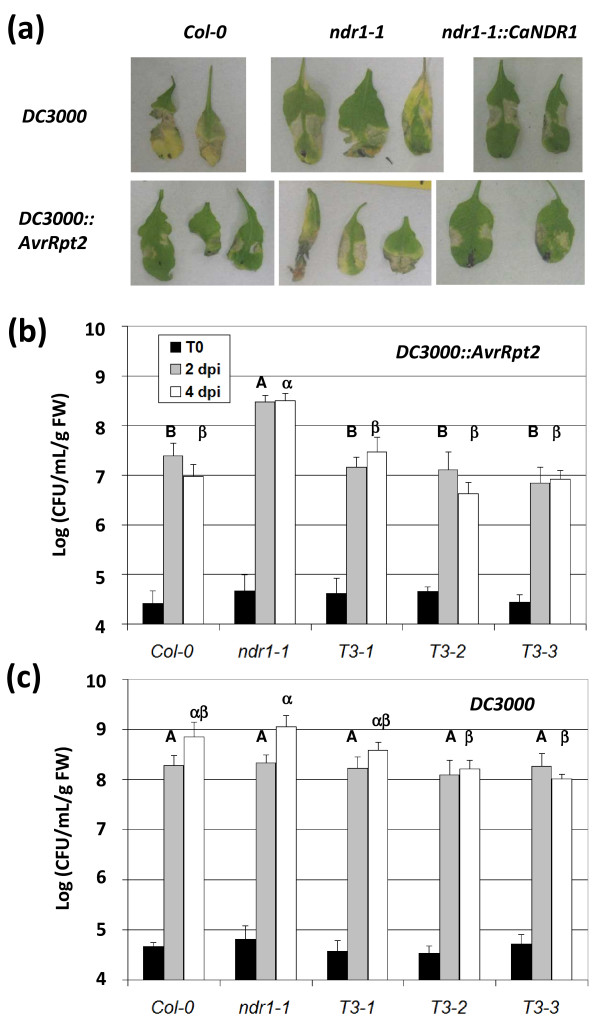
**The coffee gene *CaNDR1a *functionally complements the *Arabidopsis ndr1-1 *null mutant**. Bacterial solutions were hand-infiltrated into leaves with syringes as described in the '*Methods*' section. (a) Representative symptoms triggered by the virulent (DC3000) and avirulent (DC3000::*AvrRpt2*) *Pst *strains. A 2 × 10^7 ^cfu mL^-1 ^inoculum was used for this experiment, which was conducted twice. Pictures were taken 7 days after inoculation. (b) and (c) Bacterial growth was monitored *in planta *by assaying leaf samples 0, 2, and 4 days post-inoculation. CaNDR1a-expressing lines (T3-1, T3-2 and T3-3), like the WT plants, are resistant to *Pst DC3000*::*AvrRpt2*, whereas *ndr1-1 *mutants are susceptible. Expressing CaNDR1a in the *ndr1-1 *genetic background increased resistance to strain *DC3000*, as shown by significant reductions in leaf bacterial populations in lines T3-2 and T3-3 at 4 dpi. A 2 × 10^5 ^cfu mL^-1 ^inoculum was used for this experiment and the experiment was conducted twice. Means and standard errors (4 biological replicates) are shown for a representative experiment. Different letters indicate a significant difference at 2 dpi (Roman letters) or 4 dpi (Greek letters), as determined by ANOVA of square-root transformed data followed by a Student-Newman-Keuls (SNK) test (α < 5%). No significant difference in leaf bacterial concentration was observed among *Arabidopsis *genotypes at T0.

Previous work has shown that the *ndr1-1 *null mutant is incapable of HR activation in response to *Pst *strain DC3000::*AvrRpt2 *carrying an *AvrRpt2 *cassette-containing plasmid [20,21]. Conversely, a high overexpression level of *AtNDR1 *in the *Col-0 *genetic background was found to confer enhanced disease resistance to strain DC3000 [22]. The behavior of our overexpressor lines was thus examined in response to the two isogenic bacterial strains (DC3000::*AvrRpt2 *and DC3000) by recording macroscopic symptoms and following *in planta *bacterial growth over a four-day period. Although Coppinger *et al*. [22] had previously reported the occurrence of HR-like lesions in non-inoculated *Arabidopsis *transgenic lines overexpressing *AtNDR1*, no such lesions were observed in our non-inoculated T3 lines. Although the three genotypes developed disease symptoms in response to DC3000 (Figure 3a), T3-2 and T3-3 lines were less susceptible than the *ndr1-1 *mutant plants, as shown by the leaf bacterial contents at four days post-inoculation (dpi) (Figure 3c). Upon a challenge with DC3000*::AvrRpt2*, WT plants exhibited typical hypersensitive lesions located within the infiltrated area, whereas *ndr1-1 *mutants showed disease-like symptoms characterized by tissue yellowing, which spread outside the inoculated zone (Figure 3a). As expected, such striking differences between the WT and *ndr1-1 *genotypes were closely correlated with leaf bacterial amounts. For instance, as early as 2 dpi, mutant leaves already showed a 10-fold increase in the concentration of bacteria compared to WT leaves (Figure 3b). More importantly, when inoculated with strain DC3000::*AvrRpt2*, all three *CaNDR1a*-expressing lines presented a HR-like phenotype (Figure 3a) that was associated with bacterial levels statistically comparable to that of WT plants (Figure 3b). Furthermore, expression of the coffee transgene in the *Arabidopsis *mutant had no significant impact on the RPS4-coordinated HR that had been previously shown to be independent of *AtNDR1 *[23] (Additional file 3). Altogether, these results provide genetic evidence that *CaNDR1a *functionally and specifically complements the *ndr1-1 *mutant.

### The mature CaNDR1a protein is C-terminally processed

**Figure 4:**
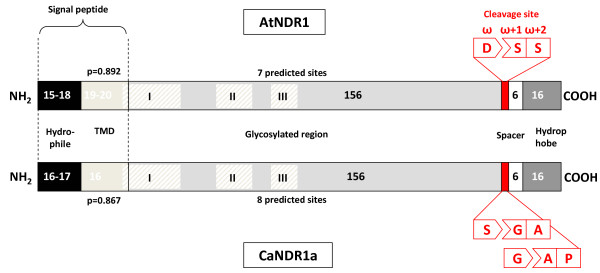
**Structural similarities between the *Arabidopsis *and coffee NDR1 proteins**. Predicted structural domains and motifs of NDR1 proteins are represented. The overall structure of both proteins appears conserved; it is furthermore reminiscent of GPI-anchored proteins [29]. The C-terminus of NDR1 proteins exhibits putative cleavage sites, including the ω-site to which the glycolipid moiety of the anchor is attached. Domains following the attachment site display the necessary features for proper transamidase activity, the enzyme complex involved in GPI modifications of proteins and localized to the ER membrane. A putative uncleavable N-terminal signal peptide that might be implicated in ER targetting is also present in both proteins. TMD indicates a predicted transmembrane domain. The size of each protein domain is indicated as Arabic numbers. The number of predicted glycosylation sites (in the middle domain, shown in light grey) is also indicated above and below the proteins. For convenience, the three conserved NHL motifs are shown as hatched regions I, II and III. Predictive models and methods used for building this scheme are described in the '*Methods*' section.

The *Arabidopsis *NDR1 protein undergoes several post-translational modifications, including multiple glycosylations and C-terminus processing. The latter cleavage removes a small portion of the protein, thereby freeing an amino-acid residue known as a ω-site (Figure 4) that was proposed to be modified by covalent binding to a glycosylphosphatidyl-inositol (GPI)-anchor [22]. In accordance with the cognate role of AtNDR1 in disease resistance signalling [23], GPI anchoring is usually encountered in eukaryotic plasma membrane-resident proteins and allows for the cell surface-tethering phenomenon [29]. Although there is no established consensus sequence of GPI-anchor attachment sites, prediction algorithms are available online. Using the Big-Pi Plant Predictor [30,31], we identified two putative overlapping cleavage sites in the primary amino-acid sequence of CaNDR1a (Figure 4), with residues S189 and G190 being strong ω-site candidates (with *P*-values of 2.48 × 10^-6 ^and 2.76 × 10^-5^, respectively). Furthermore, CaNDR1a and its *Arabidopsis *ortholog share common structural features that are believed to be necessary for GPI attachment by the transamidase complex in endoplasmic reticulum (ER) membranes [31]. Directly downstream of the potential ω-residues is a region predicted to encompass a short polar spacer, followed by a hydrophobic tail. An uncleavable signal peptide (1-39) comprising a potential transmembrane domain (16-32) was also predicted with a high probability of occurrence (*P *= 0.867) using SignalP-3.0 software [32,33]. As previously suggested [22], this N-terminal signal sequence might be required for the protein to enter the ER network and travel through the secretory pathway.

**Figure 5:**
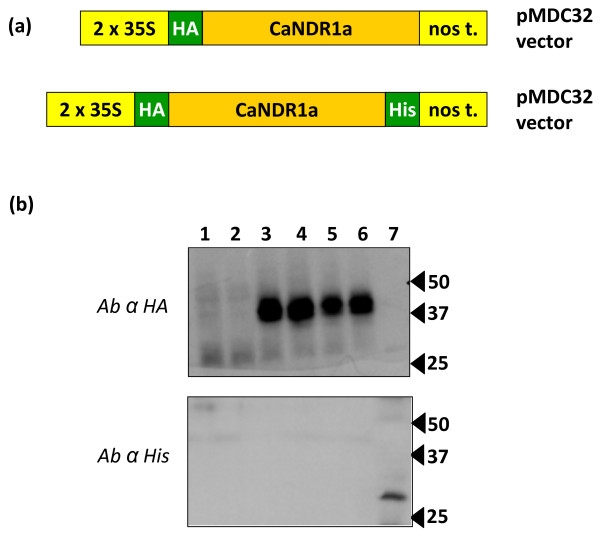
**The C-terminal end of CaNDR1a is removed from the mature protein in tobacco**. (a) Constructs used for transiently expressing HA- and HA-His-tagged CaNDR1a proteins in tobacco leaves. (b) Detection of CaNDR1a-tagged proteins by immunoblotting. The upper and lower panels show scanned films corresponding to membranes blotted with anti-HA and anti-His sera, respectively. For comparison, the same protein extracts were resolved by SDS-PAGE and subsequently transferred onto both membranes. Ten micrograms of proteins were loaded in each lane. Samples contained the main insoluble proteins that were extracted using SDS as described in the '*Methods' *section. Lanes 1 & 2, negative controls (samples prepared from leaves expressing a GUS protein and non-infiltrated leaves, respectively); lane 3, HA-positive control, His-negative control (sample prepared from tissues expressing the N-terminally HA-tagged CaNDR1a protein); lanes 4-6, samples prepared from tissues expressing the doubly-tagged CaNDR1a protein (3 independent experiments); and lane 7, HA-negative control, His-positive control (sample prepared from *Arabidopsis *leaves constitutively expressing the C-terminally His-tagged AtBI1 protein) [56].

Based on this *in silico *analysis, we decided to investigate the possibility of C-terminus processing for CaNDR1a. To this end, a doubly-tagged CaNDR1a version (HA-CaNDR1a-His) was created (Figure 5a) and transiently expressed in tobacco leaves. We reasoned that, if the CaNDR1a protein is cleaved in tobacco cells, the loss of its C-terminus should be easily visualized upon immunoblotting by the absence of a His-specific signal, whereas the proof that the protein is synthesized would be provided by the presence of a HA-specific signal.

Two to three days post-infiltration with an *Agrobacterium *strain, which was dedicated to the expression of the HA-CaNDR1a-His construct, protein extracts prepared from fresh tissues were resolved by SDS-PAGE and immunoblotted using either HA- or His-specific antisera as described in the '*Methods*' section. Immunoblot conditions were tested using a N-terminally HA-tagged CaNDR1a (HA-CaNDR1; Figure 5a) and C-terminally His-tagged Bax Inhibitor 1 (BI1-His) versions as controls. Six independent experiments including independent *Agrobacterium *infiltrations and protein extractions were carried out. Using anti-HA antibody, only one major band was detectable in lanes loaded with NDR1 samples (Figure 5b, lanes 3-6), whereas no specific signal was visualized in lanes loaded with negative control samples (Figure 5b, lanes 1, 2 & 7). Although the nucleotide sequences of *HA-CaNDR1a *and *HA-CaNDR1a-His *code for proteins with predicted molecular weights averaging 25-26 kDa, the detected proteins migrated to approximately 45 kDa under denaturating conditions. Such an apparent discrepancy is not surprising based on previous work. Coppinger and coworkers [22], indeed, showed that the native AtNDR1 protein resolved by SDS-PAGE displays a mass of about 48 kDa instead of the predicted 24.6 kDa. These authors further demonstrated that the protein regains its theoretical size when translated *in vitro *without the machinery dedicated to glycosylation, indicating that the latter post-translational modification could account for the migration shift of the mature proteins on polyacrylamide gels. Consistently, the CaNDR1a protein, like its *Arabidopsis *ortholog, exhibits a significant number of putative glycosylation sites (Figure 4). Hence, one can assume that our protein extracts (Figure 5b, lanes 3-6) are likely to contain glycosylated forms of CaNDR1a, the migration behavior of which is altered on polyacrylamide gels.

Finally, using the same set of samples and anti-His antibody, we were unable to detect HA-NDR1-His protein (Figure 5b, lanes 4-6), whereas BI1-His protein (31 kDa) was clearly identified (Figure 5b, lane 7). The latter data indicate that CaNDR1a is C-terminally processed in tobacco leaves, which strongly suggests that the protein is modified by addition of a GPI moiety. Further experiments are nevertheless needed to confirm this assumption.

### CaNDR1a is localized to the plasma membrane

**Figure 6:**
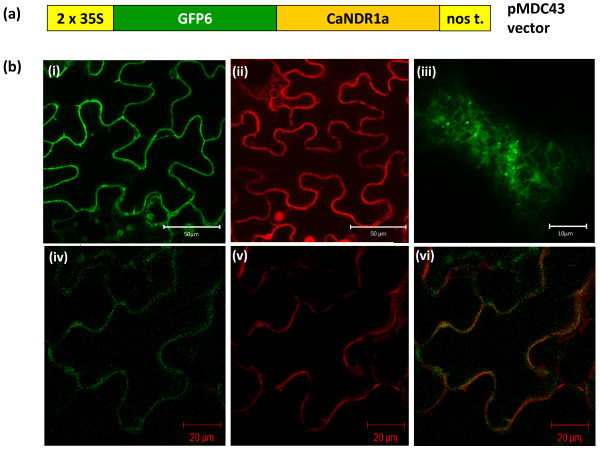
**The CaNDR1a protein is localized at the plasma membrane**. (a) Scheme of the construct used for determining the subcellular localization of CaNDR1a protein. (b) Confocal-laser microscopy pictures illustrating the plasma membrane localization of CaNDR1a: (i) GFP6-CaNDR1a; (ii) mCherry-labeled protein targeted to the plasma membrane; (iii) GFP6-CaNDR1a, a close-up of the internal labeling observed in a few cells; (iv), (v) and (vi), colocalization experiments where both the GFP6-CaNDR1a and mCherry-labeled plasma membrane marker were simultaneously expressed in the same cells. Independent experiments were conducted five times.

Indirect data support the association of the CaNDR1a protein with membranes: (i) the potential post-translational modification by addition of a GPI-anchor; (ii) a predicted transmembrane-spanning domain located within the N-terminal signal peptide (Figure 4), and (iii) the need of a detergent for the protein to be extracted from tobacco leaf tissues when transiently expressed (Additional file 4). Accordingly, the CaNDR1a protein was predicted to be localized to the plasma membrane (PM) using ChloroP1.1 and PSORTII software [34,35]. Therefore, in order to assess its subcellular localization, a GFP6 translational fusion was created (Figure 6a), transformed into leaf epidermal tobacco cells using *Agrobacterium tumefaciens *as the vector, and imaged by confocal microscopy (as described in the '*Methods*' section). In accordance with our working hypothesis, independent experiments showed a consistent fluorescent pattern delineating cellular contours (Figure 6b, panel i). Such a pattern was also observed (Figure 6b, panel ii) with a PM-resident protein fused to mCherry fluorophore [36]. In addition, further experiments where both proteins were simultaneously expressed in the same cells revealed a significant overlap between the GFP6 and mCherry signals at the cell surface (Figure 6b, panels iv, v, vi). It is noteworthy that a few GFP6-CaNDR1a-expressing cells displayed not only cell surface labeling, but also internal fluorescence resembling an ER-like reticulated network with brighter dots that could represent Golgi structures (Figure 6b, panel iii).

**Figure 7:**
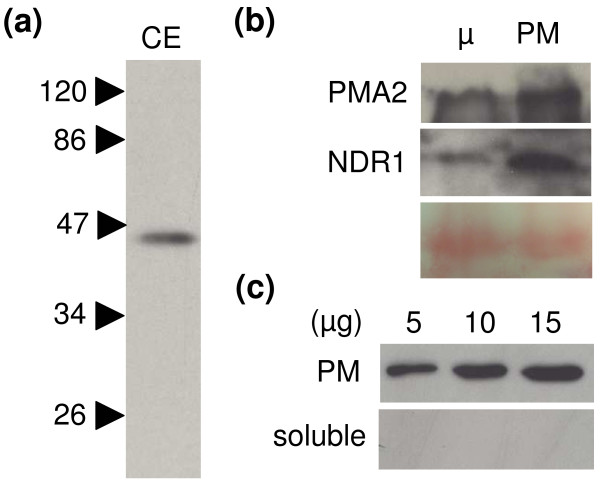
**The CaNDR1a protein is enriched in plasma membrane fraction**. The HA-CaNDR1a construct (see Figure 5a) was used for carrying out two independent experiments that consisted of two independent agroinfiltrations and plasma membrane (PM) preparations. A representative experiment is presented in this figure. Agroinfiltration and immunoblot conditions are described in the '*Methods*' section. (a) Detection of HA-CaNDR1a proteins in *Agrobacterium*-infiltrated leaf tissues. Crude extract (CE) was prepared by directly incubating tissues at 95°C for 5 min in 1X Laemmli buffer [57]. (b) Detection of HA-CaNDR1a proteins and endogenous PM-resident proteins PMA2 in microsomal and PM fractions. PMA2 is a proton-ATPase pump previously shown to be localized exclusively at the PM [61]. Membrane was probed using a specific anti-PMA2 serum [58] in order to check for the purity of the PM fraction. As expected, PMA2 proteins appeared to be significantly enriched in the PM fraction versus the microsomal (μ) one, as also observed for HA-CaNDR1a proteins upon stripping and reprobing of the same blotting membrane with HA-specific antiserum (Middle panel). Membranes were also stained with Ponceau S to show the equal loading between both fractions, i.e. μ and PM (lower panel). (c) HA-tagged CaNDR1a proteins are not detected in soluble fractions. Distinct protein amounts of soluble (100.000 × g supernatant) and PM fractions (5, 10 and 15 μg) were resolved by SDS-PAGE and immunoblotted using a HA-specific antiserum.

Because leaf epidermal tobacco cells possess a large central vacuole that presses the cytoplasmic compartment against the PM and cell wall, it is difficult to conclude on the subcellular localization of CaNDR1a based solely on our microscopy data. In order to unambigously ascertain the localization of CaNDR1a, the N-terminally HA-tagged version of CaNDR1a (Figure 5a) was transiently expressed in tobacco leaves and purified PM fractions were directly tested for the presence of the protein by immunoblotting using HA-specific antisera. Immunoblotting of crude extracts (CE) prepared by directly boiling agroinfiltrated tissues in Laemmli buffer indicated that HA-CaNDR1a proteins were succesfully expressed in plant cells (Figure 7a). Most importantly, the tagged version of CaNDR1a was significantly enriched in PM fractions compared to microsomal ones, as also observed for the endogenous PM-resident protein PMA2 (Figure 7b). In addition, while no signal was detected when 5, 10 and 15 μg proteins of the soluble fraction (100.000 × g supernatant) was blotted, a HA-specific band, the intensity of which increased with the amount of total proteins loaded, was clearly visualized (Figure 7c). Altogether, these results show that the mature CaNDR1a protein is targeted to PM in the tobacco heterologous system, further suggesting a similar subcellular localization for the protein in coffee cells.

### Identification of a potential homologous RIN4 protein from coffee plants

**Figure 8:**
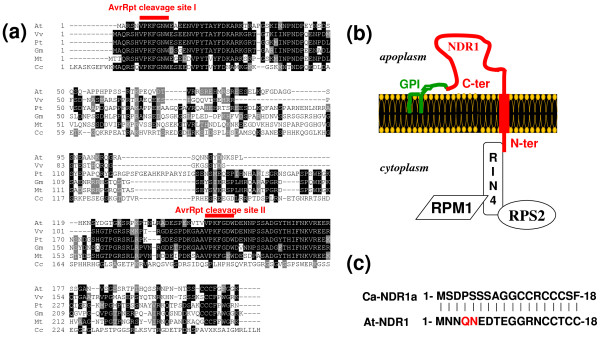
**Putative mechanistic conservation of NDR1 function**. (a) Alignment of RIN4 homologous sequences. The closest sequence homologs of AtRIN4 [AGI:At3g25070] were aligned with the putative coffee RIN4 protein [harvEST:Coffea:UG5351] using ClustalX [59]. The positions of the two AvrRpt2 cleavage sites [37,38] are highlighted in red. Accession numbers of the genes coding for the proteins presented in the figure are as follows: *Glycine max *Gm [GenBank:ADJ67468]; *Medicago truncatula *Mt [GenBank:ACJ83941]; *Populus trichocarpa *Pt [GenBank:XP_002301798]; *Vitis vinifera*, Vv [GenBank:CBI33050]. (b) Scheme showing how NDR1 is anchored to the plasma membrane. AtNDR1 indirectly retains both R-proteins, RPS2 and RPM1, at the plasma membrane *via *its interaction with RIN4 [24,48]. (c) Comparison of the N-terminal portions of the two orthologous NDR1 proteins from *A. thaliana *and *C. arabica*. Amino-acid residues necessary for the interaction with AtRIN4 are highlighted in red. Intriguingly, these residues do not seem to be conserved in the coffee sequence.

The *Arabidopsis *NDR1 protein has been demonstrated to physically interact with RIN4 both in a yeast heterologous system and *in planta *[24]. Searching for RIN4 sequence homologs in the HarvEST^© ^Coffea database resulted in the identification of a candidate contig from *Coffea canephora *[GenBank: DV705409.1]. The deduced protein sequence shares a high percentage of identity/homology (36/53%) with the beginning of our query sequence, AtRIN4. This region is also highly conserved within the RIN4 family of proteins (Figure 8a). One of the two cleavage sites that permit the hydrolysis of RIN4 upon delivery of the bacterial protease AvrRpt2 into *Arabidopsis *cells [25,37,38] is also conserved in the coffee protein (Figure 8a). In line with our previous data (Figures 5, 6 and 7), this *in silico *analysis points to potential mechanistic conservation of the NDR1 function in *Arabidopsis *and coffee plants.

## Discussion

The *Arabidopsis ndr1 *locus was identified in the late 1990's using a forward genetic screen based on the loss of resistance to the *Pst *strain DC3000::*AvrRpt2 *[20,21]. Since then, *NDR1 *homologous genes have been found by sequence comparison in other plant species such as *Brassica napus *[39] and *Vitis vinifera *[40]. Many sequence homologs (around 19 non-redundant hits within 11 plant species) can also be retrieved from the GenBank database by means of the BLAST P algorithm (data not shown). However, to our knowledge, our data constitute a novel report on the identification and characterization of a functional *NDR1 *homolog, despite the plethora of orthologous candidates.

In this study, several lines of evidence indeed demonstrated that ectopic expression of *CaNDR1a *coding sequence was able to rescue the phenotype of the *Arabidopsis ndr1-1 *null mutant. Upon infection with DC3000::*AvrRpt2*, the three mutant lines expressing the coffee transgene were found to develop hypersensitive cell death symptoms that were absent in mutant plants (Figure 3a). This macroscopic study was further corroborated by two independent *in planta *bacterial growth assays showing that leaf populations of the bacterial pathogen in our transgenic lines were low and comparable to those of WT plants (Figure 3b). In addition, high overexpression level of the coffee *CaNDR1a *gene in the *Col-0 *genetic background was also found to confer enhanced disease resistance to the DC3000 strain, as previously reported when the *AtNDR1 *gene was overexpressed in *A. thaliana *[22].

Importantly, NDR1-driven resistance in *A. thaliana *is not restricted to bacterial pathogen attacks. Two reports have demonstrated that the *ndr1 *mutation renders plants susceptible to infection by the oomycete *Hyaloperonospora arabidopsidis *[20] and the fungus *Verticillium longisporum *[41]. Therefore, given that (i) *CaNDR1a *is a functional homolog of the *Arabidopsis NDR1 *gene, and (ii) transcripts of the former accumulate in coffee leaves undergoing HR in response to the fungus *H. vastatrix *[16,19], it would not be surprising if NDR1 proteins could regulate the defense signaling pathway(s) leading to coffee rust resistance. This hypothesis is currently under investigation in our laboratory using a functional approach. Recently, we also showed that *A. thaliana Col-0 *plants display a rapid non-host response to *H. vastatrix*. This response is reminiscent of HR in that it prevents haustorium formation and hyphal spread in plant tissues [42]. This work raises the possibility of testing the role of NDR1 in response to the coffee leaf rust in the *A. thaliana *heterologous system.

As predicted by our bioinformatic analysis, imaging of GFP6-tagged CaNDR1a protein by confocal microscopy revealed a fluorescent pattern that was consistent with a plasma membrane localization (Figure 6b, (i)). Colocalization experiments with a PM fluorescent protein marker also supported this observation (Figure 6b, (iv-vi)). Furthermore, the need of an anionic detergent like sodium dodecyl-sulfate for the HA-tagged CaNDR1a proteins to be extracted from tobacco leaves (Additional file 4) indicated an association with membranes. Finally, our biochemical approach based on the purification of PM by two-phase PEG/dextran partitioning (Figure 7b,c) clearly demonstrated the presence of HA-CaNDR1a proteins in tobacco PM fractions. Therefore, it is likely that the mature CaNDR1a protein resides in the plasma membrane of coffee cells.

No fluorescent labeling of the organelle corresponding to a GFP6 spectrum was observed in chloroplasts, although it had been reported previously for a tagged version of AtNDR1 [27]. Instead, internal reticulated labeling reminiscent of the ER network (Figure 6b, (iii)) was observed in a few cases and may correspond to cells overloaded with the ectopic fluorescent proteins. This observation is consistent with our results, suggesting that the CaNDR1a protein could be modified by addition of a GPI moiety to its C-terminal part (Figure 5b). It has been well-described that proteins tethered to the cell surface by means of a GPI anchor undergo this sort of post-translational modification in the ER before being sorted *via *the secretory pathway to their final destination, i.e., the plasma membrane.

Usually, GPI-anchored-proteins are also thought to locate on the apoplasm side of the plasma membrane [43]. In *A. thaliana*, it has been clearly established that NDR1 is attached to the plasma membrane through a C-terminal GPI anchor [22]. It has also been inferred that the N-terminal portion of NDR1 lies within the cytoplasm because it was found to interact with the cytosolic protein RIN4 *in planta *[24]. Since the C-terminal anchor of AtNDR1 is resistant to cleavage by phospholipase C, these data further led to the hypothesis that the protein possesses a transmembrane-spanning domain as a second anchor site. This was recently corroborated by a modelling study **[**44**] **and, in fact, the coffee protein, like its *Arabidopsis *relative, was predicted to present a single transmembrane domain (Figure 4), suggesting a similar, but atypical topology of the two counterparts (Figure 8b).

Recently, a new mode of action of NDR1 was revealed by Knepper *et al*. [44]. Based on structural homology with mammalian integrins and the *Arabidopsis *late embryogenesis abundant (LEA) protein 14, known to be involved in abiotic stress response [45], the aforementioned authors investigated the possibility that AtNDR1 may control cell integrity through PM-cell wall adhesions. Besides its well-characterized role as a key signaling component during pathogen attack, a broader function for NDR1 is strongly suggested by the data in mediating primary cellular functions in *Arabidospsis *through maintenance of PM-cell wall connections [44]. From these unexpected results, the question arises as to whether or not CaNDR1a could perform a similar function in *C. arabica*.

Interestingly, upon inoculation with DC3000::*AvrRpt2*, successful activation of HR required NDR1-RIN4 physical interaction. Further examination using an alanine-scanning mutagenesis strategy revealed that two amino acid residues within the N-terminal part of NDR1 were necessary for the interaction [24]. Despite the apparent lack of conservation of these two amino acid determinants within the CaNDR1a end (Figure 8c), our results showing that the coffee gene was able to restore RPS2-mediated resistance in the *ndr1-1 *mutant tend to prove that CaNDR1a does interact with AtRIN4 in our transgenic lines. Thus, this raises the possibility that mechanism(s) whereby NDR1 proteins exert their function could be conserved in *Arabidopsis *and coffee plants.

Consistent with this idea, searching for RIN4 sequence homologs in the HarvEST^© ^Coffea database resulted in the identification of a candidate contig from *Coffea canephora*. The deduced protein shows, within its N-terminal portion, a highly conserved region with the members of the RIN4 family, as well as a putative conserved canonical AvrRpt2 cleavage site (Figure 8a). Nonetheless, further experiments are needed to answer the question as to whether or not CaNDR1a, like its *Arabidopsis *ortholog, could serve as a PM anchor that indirectly recruits R-protein(s) *via *its interaction with RIN4-like intermediates (Figure 8b) [24,46]. Split-ubiquitin and yeast two-hybrid systems, combined with bimolecular fluorescence complementation (BiFC), would be useful tools for tackling this question. This might also be a faster and more convenient strategy, as opposed to classical genetic approaches, for the isolation of *R*-gene(s) conferring resistance to *H. vastatrix*. To date, no coffee *R-*gene(s) have been isolated despite the efforts of the coffee research community [3,4]. The reproductive barriers affecting genetic exchanges between diploid coffee species and the allopolyploid *C. arabica *have thus far prevented the successful isolation of the loci responsible for resistance to *H. vastatrix *through a map-based cloning strategy [4].

## Conclusions

The functional and biochemical characterization of the orthologous NDR1 protein from *C. arabica *that we have carried out represents a crucial step towards the elucidation of the molecular events underpinning resistance to coffee rust. It should help identify new players in the coffee *NDR1*-dependent signaling pathway(s) in the near future, and might thus be crucial for the engineering of transgenic coffee plants with broad spectrum resistance to *H. vastatrix *races. The development of efficient techniques to transform and propagate coffee varieties renders these biotechnological approaches feasible [47,48].

## Methods

### Plants and growth conditions

Tobacco plants (*Nicotiana benthamiana*) that were used for transient expression experiments were grown in a greenhouse, at 150 μmol/m^2^/s light radiance, with a 14/10 h, 23/20°C light-dark cycle, and 60% relative humidity.

Wild-type *Arabidopsis thaliana *ecotype Columbia (*Col-0*), *ndr1-1 *null mutants [20], and transgenic lines expressing *CaNDR1a *were all grown in a growth chamber under short-day conditions (10 h photoperiod, 100 μmol/m^2^/s light fluency), at 22/20°C day/night with 80% relative humidity. Pathogen challenge conditions are described hereafter.

### Isolation and cloning of CaNDR1a/b cDNA

As previously described [19], the 5' end of the *CaNDR1 *cDNA that is referred to as DSS12 had already been sequenced (Genbank:CO773976). 3'-RACE PCR was thus conducted to determine the sequence of the full-length cDNA. Total RNA (1 μg) isolated from *C. arabica *cv. *caturra *leaves that had been challenged with *H. vastatrix *for 18 hours were first reverse-transcribed using the Smart CDS primer and a combination of the Omniscript RT (Qiagen, Courtaboeuf, France) and SMART PCR cDNA synthesis kits (Clontech, Mountain View, CA, USA). RACE assays were then performed using specific oligonucleotides designed in the 5' non-coding region (3R-NDR1, 5'-CTACTTTGTTCACTGGTAGTCCCTC-3'; n3R-NDR1, 5'-CATAATACTTCACCGGAGAACCACC-3') and the 5'PCR Smart primer (Clontech). The resulting 1-kb PCR product was cloned into the pGEM-Teasy vector (Promega, Charbonnières-les-bains, France) and finally sequenced (Genome Express, Grenoble, France).

### Constructs

To assess the complementation of the *Arabidopsis *null mutant *ndr1-1 *[20], the open reading frame of CaNDR1a was cloned into the binary vector pCAMBIA 1305.1 (Cambia, Brisbane, Australia) downstream of the strong and constitutive 35S promoter of the cauliflower mosaic virus. For this purpose, the *iud *gene was removed from the vector by restriction digestion with *BglII *and *BstEII *enzymes. The coding sequence of *CaNDR1a *was amplified by PCR (DAP Goldstar DNA polymerase, Eurogentec, Seraing, Belgium) using the corresponding pGEM-T clone (GenBank:DQ335596) as a template and the following primers: CaNDR1-*Bgl*II 5'-TCAGATCTTATGGACAAAGGATGGGGC-3', and CaNDR1-*BstE*II 5'-TAGGTCACCAAATTAATTCCCAGGAAA-3'. Digested PCR products were then ligated into the binary vector to get the final construct.

To test the hypothesis that the C-terminal part of CaNDR1a is removed from the mature protein, single- and double-tag constructs were created (Figure 5). *CaNDR1a *was amplified by PCR using a high fidelity DNA polymerase according to the manufacturer's instructions (*PfuTurbo*, Stratagene, La Jolla, CA, USA). The following primer couple was used for directly adding haemaglutinin (HA) and poly-histidine (His) sequences to the 5'- and 3'-ends of the PCR products, respectively: CaNDR1-Forward 4, 5'-CACC ATG TAT CCC TAC GAC GTA CCA GAT TAT ATG TC AGA CCC CAG CAG CAG TGC-3' and CaNDR1-Reverse 3, 5'-CTA ATG GTG ATG GTG ATG GTG CAA CAG CAG AAC CAA GAA A-3'. The primers used for obtaining the single HA-tagged version were CaNDR1-Forward 4 and CaNDR1-Reverse 2 (5'-CTA CAA CAG CAG AAC CAA GA-3'). PCR fragments were then subcloned into the pENTR-D/TOPO vector (Invitrogen, Cergy Pontoise, France) and sequenced. To get directional cloning, the underlined nucleotide sequence was added to the forward primers. Selected clones were digested with *Mlu*-I restriction enzyme (R0198L, NEB, OZYME, Saint Quentin Yvelines, France) before overnight recombination with the binary vector pMDC32 [49] using the LR Clonase II kit (Invitrogen).

The N-terminally GFP6-tagged version was produced to examine the subcellular localization of CaNDR1a. PCR products were amplified, subcloned, sequenced and recombined with the binary vector pMDC43 [49] as described above. Primers used for the initial PCR step were as follows: CaNDR1-Forward 1, 5'-CACC ATG TCA GAC CCC AGC AGC AGT-3' and CaNDR1-Reverse 2. The vector used for *in planta *expression of the plasma membrane fluorescent marker (mCherry-tagged protein) was purchased from the Arabidopsis Biological Resource Center (ABRC) at Ohio State University (Stock # CD3-1008) [36].

Final binary constructs were all sequenced (Genome Express) prior to transformation into *Agrobacterium tumefaciens *by heat shock or electroporation methods. Bacterial strain GV3101 was used for transformation of *Arabidopsis *plants by floral dipping according to [50]. Strain LBA1119 was used for transient expression experiments in tobacco plants.

### Pathogen challenge and growth curve assays

The *Pseudomonas syringae *pv. *tomato *(*Pst *) strain *DC3000 *and the isogenic strains expressing the bacterial effector proteins AvrRpt2 or AvrRps4 were provided by Dr. Jane Glazebrook (University of Minnesota) [51]. For pathogen challenges, bacteria were grown overnight at 28°C under mild shaking in liquid King B medium. Pst DC3000 bacteria were selected with rifampicin (50 μg mL^-1^); DC3000::*AvrRpt2 *and *Pst *DC3000::*AvrRps4 *with rifampicin and tetracycline (10 μg mL^-1^). Bacteria were collected by centrifugation and resuspended at 2 × 10^5 ^CFU mL^-1 ^in physiological water (9 g NaCl/L) prior to inoculation.

Progeny of *Arabidopsis ndr1-1 *T0 plants (*ndr1-1::CaNDR1a*) were screened on half-strength Murashige and Skoog medium supplemented with 30 μg mL^-1 ^hygromycin. Transformation of individual resistant seedlings was confirmed by PCR using genomic DNA as the template and *CaNDR1a-*specific primers (CaNDR1-*Bgl*II and CaNDR1-*BstE*II). Homozygous single locus insertion lines were then isolated by following segregation of hygromycin-resistant plants in T2/T3 generations (Additional file 2). To assess *ndr1-1 *complementation, three independent T3 lines displaying distinct expression levels for *CaNDR1a *(designated T3-1, T3-2 and T3-3) were challenged with *Pst *and *in planta *bacterial growth was followed over a four-day period (0, 2 and 4 dpi). Wild type (*Columbia, Col-0*) and *ndr1-1 *plants were also inoculated for comparison. Negative controls were infiltrated with physiological water. Half-leaves (6 to 7-week-old plants) were hand-infiltrated with a 1-mL needleless syringe. Two independent experiments that gave similar results were carried out. Each experiment comprised four replicates that were each performed by different individuals. In one replicate, each plant genotype (5 plants/genotype) was infiltrated with water or suspensions of *Pst *DC3000, *Pst *DC3000::*AvrRpt2 *or *Pst *DC3000::*AvrRps4*. Upon infiltration, plants were immediately placed in a tray covered with a plastic dome that was removed at 24 hours post-inoculation. Bacterial growth was monitored as follows. At each time point, two leaves (per plant) were harvested and ground with a mortar and pestle. The resulting mixture was serially diluted in sterile physiological water and plated onto solid King B medium supplemented with the appropriate antibiotics. The bacterial population was scored two days upon plating. Inoculation data were square-root transformed prior to ANOVA and subsequently subjected to the Student-Newman-Keuls multiple comparison test. When transformation failed to satisfy assumptions of normality and homoscedasticity, the non-parametric Kruskal-Wallis test was used.

Hypersensitive and disease symptoms were also visually assessed in an independent experiment using higher concentrations of bacterial suspension (2 × 10^7 ^CFU mL^-1^) for infiltration. Samples from this experiment were also harvested for RT-qPCR analysis.

### RNA extraction, reverse transcription and real time quantitative-polymerase chain reaction

Expression of *AtNDR1 *and *CaNDR1a *was measured as previously described [15] with the specific primers (Additional file 5) that were previously used [42]. Each assay was conducted in duplicate and included a negative control without template. The strong and constitutive actin gene (At3g18780) was chosen as internal control for normalization. Specificity of amplification was estimated by analyzing melting-temperature curves. Calculations for gene expression quantification were carried out using the comparative cycle-threshold method, as described previously [16].

### Agrobacterium tumefaciens*-mediated transient expression*

Ten mL *Agrobacterium *cultures were grown overnight under mild shaking at 30°C in regular Luria-Bertani medium containing 25 μg mL^-1 ^rifampicin, and 50 μg mL^-1 ^kanamycin when necessary. Bacteria were collected the following day by centrifugation. Pellets were resuspended in induction buffer (20 mM MES pH5.5, 10 mM MgSO4, 200 μM acetosyringone) so that OD_600 nm _of the solution reaches 0.5-0.6. Upon incubation at room temperature for 3 hours, the bacterial suspension was infiltrated onto the abaxial side of *Nicotiana benthamiana *leaves (4 to 6-week-old plants) using a needleless syringe. Samples for western blot analysis and microscopy studies were harvested 2-3 dpi. Each experiment included a transformation control that was carried out by infiltrating a bacterial clone containing a *35S::uidA intron *construct [52]. Histochemical beta-glucuronidase (GUS) staining was performed according to [53] using X-Gluc (5-bromo-4-chloro-3-indolyl-beta-D-glucuronic acid) as substrate.

### Protein colocalization by confocal microscopy

Subcellular localization of CaNDR1a was assessed by means of a transient expression system as described in the above sections. Overnight grown bacterial suspensions (GFP6-fused CaNDR1a and mCherry-fused marker) were individually induced and then mixed at 1:1 ratio before infiltration into tobacco leaves. Induction buffer and individual bacterial suspensions were also infiltrated as controls. Two to three days post infiltration, leaf disks (1.2 cm diameter) were punched from the infiltrated area and directly observed with a LSM 510 Meta Zeiss upright laser scanning confocal microscope (Objective C-Apochromat 40X/1,2 water, 488 nm laser and 505-530 band-pass filter to GFP, 543 nm laser and 585-615 band-pass filter to mCherry). Spectral imaging was obtained with a 488 nm laser on the Meta detector. After Lambda stack acquisition between 500 and 640 nm, the Linear Unmixing Function of confocal microscope discriminates between the fluorescence of GFP and mCherry in cells from reference spectra of these molecules obtained on leaves from GFP or mCherry plants (method of Emission Fingerprinting from Zeiss). The autofluorescence of chlorophyll was detected via a 650-nm long pass filter. The images were coded green (GFP) or red (mCherry). The experiment was repeated five times (each replicate included at least two infiltrated leaves per plant and three independent plants).

### Plasma membrane purification

To unambigously determine the subcellular localization of CaNDR1a proteins, the HA-tagged version of CaNDR1a was ectopically expressed in *N. benthamiana *leaves under conditions described above. Plasma membrane was prepared from infiltrated leaves at 2 dpi and purified by two-phase PEG/dextran partitioning, as previously described [54]. The purity of PM fractions was checked by assessing the enrichment of the endogenous PM-resident protein PMA2. Western blotting conditions for PMA2 are described in the next section.

### Protein extraction, SDS-PAGE and immunoblotting

Protein samples were isolated by a two-step extraction protocol. Briefly, frozen leaf tissues (1 g fresh weight) were ground in ice-cold buffer 100 mM, pH 8.0, Tris buffer (50 mL) containing 1 mM ethylenediamine tetraacetic acid, 1 mM dithiothreitol and a protease inhibitor cocktail (1 tablet for 100 mL of buffer, Complete Mini, Roche Diagnostics, Meylan, France). Mixtures were centrifuged for 40 min at 12,000 × g at 4°C. Protein concentration of supernatants was determined according to [55] using BSA as a standard. Overnight acetone precipitation was performed in order to concentrate samples. Upon western blot analysis, these crude extracts comprising the main soluble proteins appeared to contain neither of the two HA-tagged CaNDR1 versions. Mono- and polytopic membrane proteins were then extracted by resuspending the pellet in 400 μL of extraction buffer in the presence of 2% (w/v) sodium dodecyl sulphate (SDS) (Additional file 4). Mixtures were warmed in a water bath at 70°C for 15 min and centrifuged for 25 min at 18,000 × g at room temperature. Pellets were discarded. Concentration of supernatant proteins was determined using the bicinchoninic acid assay (B-9643/C-2284, Sigma-Aldrich, Saint-Quentin-Fallavier, France) according to the manufacturer's instructions. Protein samples were loaded onto 12.5% polyacrylamide gels to be separated by SDS-PAGE. Proteins were transferred for immunoblot analysis by electroblotting onto nitrocellulose membranes (0.45 μm, Hybond, GE Healthcare, Saclay, France) using X Cell II™ Blot Modules (Invitrogen). Successful transfer of proteins was checked by staining with a Ponceau S solution. Membranes were then incubated overnight at 4°C under mild shaking in a Tris-buffered saline solution containing 4% (w/v) dry milk (Cat. # 170-6404, Bio-Rad, Marnes-la-Coquette, France) and 0.2% (v/v) Tween 20. They were probed with anti-HA-HRP (Cat.# A00169, GenScript Corporation, Paris, France) or anti-(His)_5_-HRP (Cat.# 34460, Qiagen) antibodies to detect epitope-tagged proteins. Both antibodies were used at a 1:2000 dilution.

Protein samples used as positive controls for His blots were prepared from *Arabidopsis *transgenic lines constitutively overexpressing a C-terminally His-tagged version of AtBI1 [56]. Seeds were kindly provided by Dr. Eric Lam (Biotechnology Center for Agriculture and the Environment, Rutgers University, USA). Proteins were extracted as described by the authors [56]. Freshly harvested leaves were directly ground in Laemmli buffer [57], warmed at 95°C for 5 min and centrifuged. The resulting supernatant was resolved by SDS-PAGE and blotted like other protein samples. The expected size of epitope-tagged AtBI1 is about 31 kDa.

Microsomal and plasma membrane samples were resolved by SDS-PAGE and transferred onto PVDF membranes for immunoblotting under the exact same conditions as other protein samples. HA-CaNDR1a detection was carried out as described above. When membranes were probed with antibodies raised against PMA2 (1:16.000 dilution) [58], a goat anti-rabbit antibody coupled to HRP (Cat.# 656120, Invitrogen) was used as secondary antibody at a 1:2000 dilution. To test for the presence of HA-CaNDR1a, membranes probed with the PMA2-specific antiserum were then stripped off in the electrophoresis SDS-PAGE migration buffer in the presence of β-mercaptoethanol (28 mM final concentration) at 50°C for 30 minutes. Membranes were then blocked and reprobed with anti-HA-HRP antibodies.

### Bioinformatic analysis

Searches for CaNDR1 sequence homologs in the GenBank database were performed by means of Basic Local Alignment Search Tools, or BLAST [27], available online at the National Center for Biotechnology Information (http://ncbi.nlm.nih.gov/). Sequences were aligned using the ClustalX algorithm (version 2.0.10) [59] and further processed online at the BoxShade server (http://www.ch.embnet.org/software/BOX_form.html). The phylogenetic tree was built using Phylowin freeware using the neighbor-joining method [60]. Putative GPI-anchor attachment sites were identified using the Big-Pi Plant Predictor (http://mendel.imp.univie.ac.at/sat/gpi/plant_server.html) [30,31]. The occurence of signal peptides and transmembrane domains within primary amino-acid sequences was assessed using SignalP-3.0 (http://www.cbs.dtu.dk/services/SignalP/) [32,33]; that of glycosylation sites was predicted using NetNGlyc 1.0 (http://www.cbs.dtu.dk/services/NetNGlyc/). The freeware Mwcalc was used for calculations of the theoretical protein molecular weight and isoelectric point (http://sourceforge.net/search/?q=mwcalc). Subcellular localization of proteins was predicted using the PSORTII program [34]. ChloroP1.1 [35] was also used for checking for the absence of putative chloroplast-targeting sequences in our proteins of interest. HarvEST^© ^software that was used to identify the coffee RIN4-like protein is available online at http://harvest.ucr.edu/.

## List of abbreviations

BiFC: bimolecular fluorescence complementation; ER: endoplasmic reticulum; EST: expressed sequence tag; GPI: glycosyl-phosphatidylinositol; HIN1: Harpin-induced gene 1; HR: Hypersensitive Response; NDR1: Non-race specific Disease Resistance 1; NHL: NDR1/HIN1-like; PCR: polymerase chain reaction; Pst: *Pseudomonas syringae *pv. *tomato*; *R*-gene: *Resistance*-gene; RACE: Rapid Amplification of cDNA ends; RIN4: RPM1-interacting protein 4.

## Authors' contributions

JLC & ASP carried out the bioinformatic analysis; JLC, ASP & JE performed the cloning experiments; JLC, SM & GC carried out the microscopy study; JLC purified the plasma membrane and conducted the western blotting approach; JLC, JE, LB & DF performed the pathogen inoculation and *in planta *growth assay; ASP conducted the RT-qPCR experiments; LB conducted the statistical analysis. JLC, LB & DF designed/interpreted the experiments. JLC & DF wrote the manuscript. All authors read and approved the final manuscript.

## Supplementary Material

Additional file 1 **Full length alignment of *CaNDR1a *coding sequence with its *Arabidopsis *relatives**. Alignment was performed as described in the legend of Figure 1. For sequence ID, see also the legend of Figure 1. The positions of the three NHL motifs within sequences are highlighted in red. Click here for file

Additional file 2 **T2 segregation results of *CaNDR1a *transgenic lines used in this study**. Table showing the segregation of Hyg^R ^and Hyg^S ^phenotypes in T2 progeny from three T1 transgenic lines of *Arabidopsis thaliana *expressing CaNDR1a. The T3 lines that were selected for further work originated from T2 individuals that gave only Hyg^R ^phenotypes upon selfing. Click here for file

Additional file 3 **Ectopic expression of *CaNDR1a *in *Arabidopsis ndr1-1 *null mutant does not alter resistance to *Pseudomonas syringae *pv. *tomato *(DC3000::*AvrRps4*)**. Inoculation experiments were carried out as described in the '*Methods*' section. A 2 × 10^-5 ^cfu mL^-1 ^inoculum was used for this experiment, and the experiment was conducted twice. Bacterial growth was measured *in planta *over a four-day period. Means and standard errors (4 biological replicates) are shown for a representative experiment. Putative differences among leaf bacterial concentrations at T0 and 4 dpi were statistically assessed by ANOVA of square-root transformed data followed by a SNK test (α < 0.05). Data measured at 2 dpi were analyzed using the non-parametric Kruskal-Wallis test. No significant differences in leaf bacterial concentration were observed among the *Arabidopsis *genotypes. Click here for file

Additional file 4 **Detergent is needed to extract CaNDR1a from tobacco leaves**. CaNDR1a-tagged proteins that were transiently expressed in tobacco leaves were resolved by SDS-PAGE and subsequently transferred onto membrane by immunoblotting. Panel shows the scanned film corresponding to a representative membrane blotted with anti-HA serum (3 independent experiments). Ten μg of protein were loaded in each lane. Samples containing the main insoluble proteins extracted using SDS were loaded in lanes 1-4; those containing the main soluble proteins extracted without SDS were loaded in lanes 5-8. Protein extracts were prepared as described in the '*Methods' *section. Lanes 1 & 5, samples prepared from tissues expressing the doubly-tagged CaNDR1a protein; lanes 2 & 6, samples prepared from leaves expressing the N-terminally HA-tagged CaNDR1a protein; lanes 3 & 7, negative controls, samples prepared from leaves infiltrated with the buffer that was used for resuspending *Agrobacterium *pellets; lanes 4 & 8, negative controls, samples prepared from non-infiltrated leaves. Click here for file

Additional file 5 **Primers used for real-time quantitative PCR approach of gene expression in *35S::CaNDR1 A. thaliana *transformed lines**. Table with the name and sequence of primers used for RT-qPCR. Click here for file
