# Supplementary material for: Identification and characterization of the Non-race specific Disease Resistance 1 (NDR1) orthologous protein in coffee
Source: BMC Plant Biol. 2011 Oct 24;11:144. doi: 10.1186/1471-2229-11-144 (PMC3212813; doi:10.1186/1471-2229-11-144)
Supplement: Additional file 1 — Full length alignment of CaNDR1a coding sequence with its Arabidopsis relatives. Alignment was performed as described in the legend of Figure 1. For sequence ID, see also the legend of Figure 1. The positions of the three NHL motifs within sequences are highlighted in red. [file 1471-2229-11-144-S1.PDF]

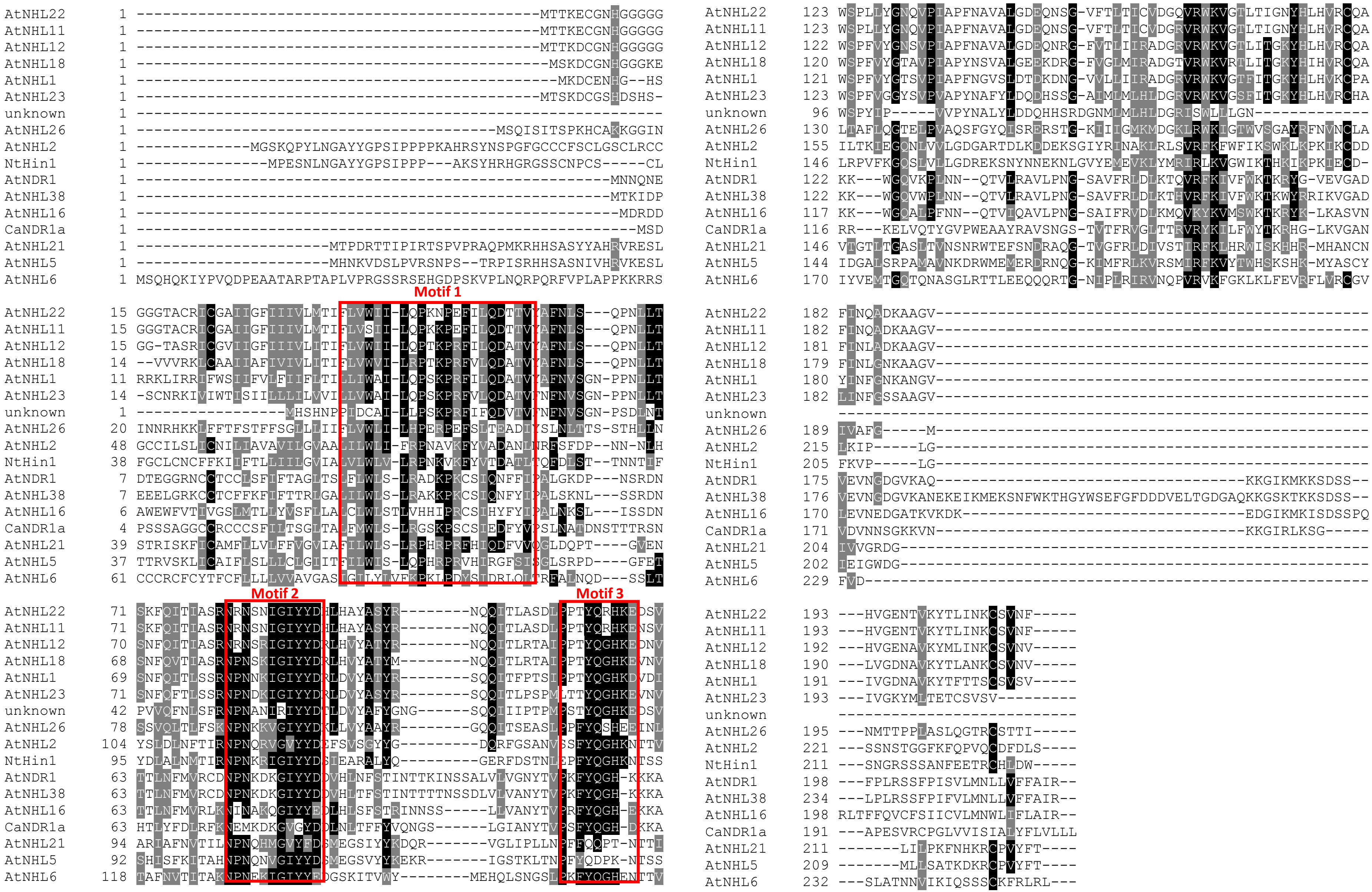
Motif 1
Motif 2
Motif 3
