# Supplementary material for: Identification and characterization of the Non-race specific Disease Resistance 1 (NDR1) orthologous protein in coffee
Source: BMC Plant Biol. 2011 Oct 24;11:144. doi: 10.1186/1471-2229-11-144 (PMC3212813; doi:10.1186/1471-2229-11-144)
Supplement: Additional file 2 — T2 segregation results of CaNDR1a transgenic lines used in this study. Table showing the segregation of HygR and HygS phenotypes in T2 progeny from three T1 transgenic lines of Arabidopsis thaliana expressing CaNDR1a. The T3 lines that were selected for further work originated from T2 individuals that gave only HygR phenotypes upon selfing. [file 1471-2229-11-144-S2.PDF]

**Population**	**Total # seedlings**	**Hyg^R**	**Hyg^S**	**Hyg^R/Hyg^S**	**Chi^2**
**T2-1**	**110**	**75**	**35**	**2.14**	**2,73**
**T2-2**	**76**	**45**	**31**	**1.69**	**7,17****
**T2-3**	**45**	**28**	**17**	**1.65**	**3,92***

* and **indicate that observed Hyg^R/Hyg^S ratios differed significantly from the expected 3:1 ratio at *P*=0.05 (critical value = 3.84) and *P*=0.01 (critical value = 6.64), respectively, according to the Chi^2 test.
