# Supplementary figures and images for: Identification and characterization of the Non-race specific Disease Resistance 1 (NDR1) orthologous protein in coffee
Source: BMC Plant Biol. 2011 Oct 24;11:144. doi: 10.1186/1471-2229-11-144 (PMC3212813; doi:10.1186/1471-2229-11-144)

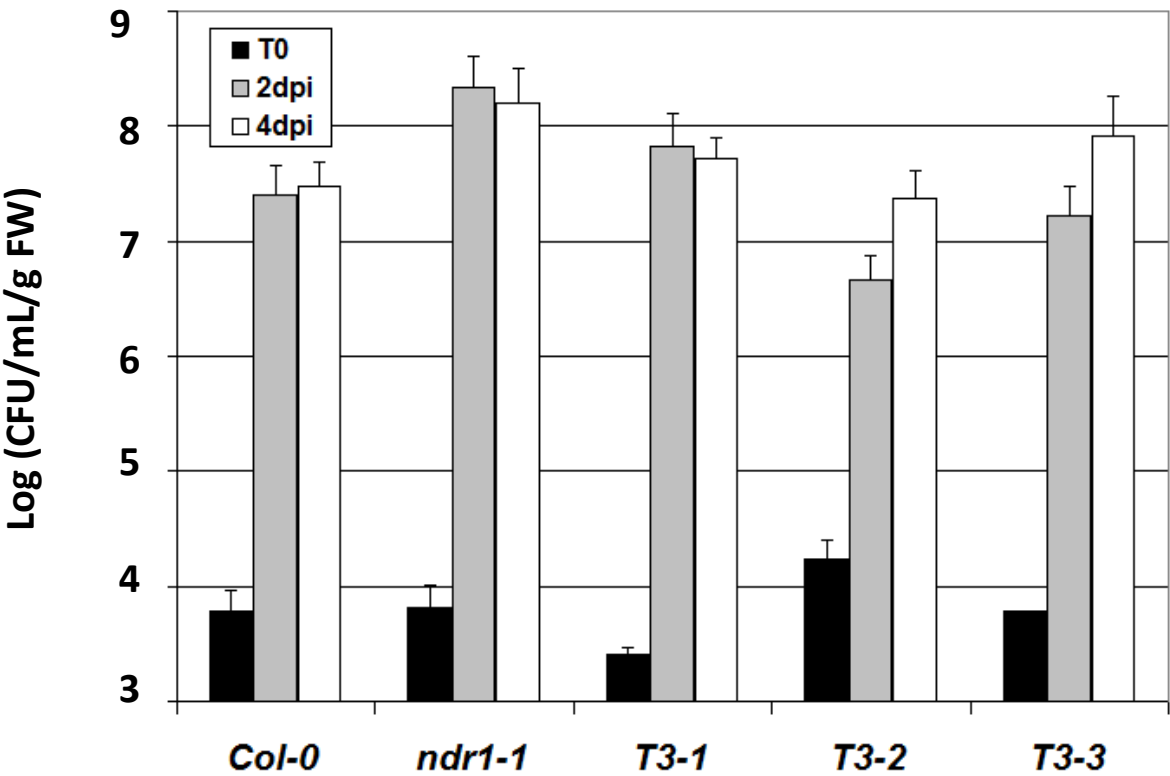

Supplement: Additional file 3 — Ectopic expression of CaNDR1a in Arabidopsis ndr1-1 null mutant does not alter resistance to Pseudomonas syringae pv. tomato (DC3000::AvrRps4). Inoculation experiments were carried out as described in the 'Methods' section. A 2 × 10^-5^ cfu mL-1 inoculum was used for this experiment, and the experiment was conducted twice. Bacterial growth was measured in planta over a four-day period. Means and standard errors (4 biological replicates) are shown for a representative experiment. Putative differences among leaf bacterial concentrations at T0 and 4 dpi were statistically assessed by ANOVA of square-root transformed data followed by a SNK test (α < 0.05). Data measured at 2 dpi were analyzed using the non-parametric Kruskal-Wallis test. No significant differences in leaf bacterial concentration were observed among the Arabidopsis genotypes. [file 1471-2229-11-144-S3.PDF]

**1 2 3 4 5 6 7 8**

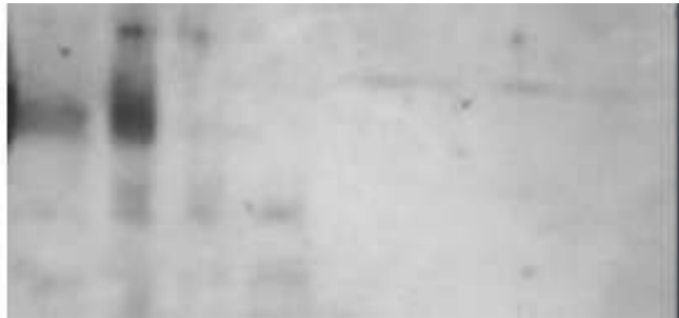

**50**

**37**

**25**

Supplement: Additional file 4 — Detergent is needed to extract CaNDR1a from tobacco leaves. CaNDR1a-tagged proteins that were transiently expressed in tobacco leaves were resolved by SDS-PAGE and subsequently transferred onto membrane by immunoblotting. Panel shows the scanned film corresponding to a representative membrane blotted with anti-HA serum (3 independent experiments). Ten μg of protein were loaded in each lane. Samples containing the main insoluble proteins extracted using SDS were loaded in lanes 1-4; those containing the main soluble proteins extracted without SDS were loaded in lanes 5-8. Protein extracts were prepared as described in the 'Methods' section. Lanes 1 & 5, samples prepared from tissues expressing the doubly-tagged CaNDR1a protein; lanes 2 & 6, samples prepared from leaves expressing the N-terminally HA-tagged CaNDR1a protein; lanes 3 & 7, negative controls, samples prepared from leaves infiltrated with the buffer that was used for resuspending Agrobacterium pellets; lanes 4 & 8, negative controls, samples prepared from non-infiltrated leaves. [file 1471-2229-11-144-S4.PDF]
