# Supplementary material for: Identification and characterization of the Non-race specific Disease Resistance 1 (NDR1) orthologous protein in coffee
Source: BMC Plant Biol. 2011 Oct 24;11:144. doi: 10.1186/1471-2229-11-144 (PMC3212813; doi:10.1186/1471-2229-11-144)
Supplement: Additional file 5 — Primers used for real-time quantitative PCR approach of gene expression in 35S::CaNDR1 A. thaliana transformed lines. Table with the name and sequence of primers used for RT-qPCR. [file 1471-2229-11-144-S5.PDF]

Gene name	Forward primer	Reverse primer
Actin (AT3G18780)	TCCCTCAGCACATTCCAGCAGAT	AACGATTCCTGGACCTGCCTCATC
NDR1 (AT3G20600)	GAAGACACAGAAGGTGGTCGAAAC	TTTTCCGAGGGCAGGAATGA
CaNDR1	GTGTTGCCGATGCTGCTG	AGTAGTGGTGGAATTATCTGTTGC
